# Supplementary material for: Investigating the profile of patients with idiopathic inflammatory myopathies in the post-COVID-19 period
Source: Microbiol Spectr. 2025 May 15;13(6):e00134-25. doi: 10.1128/spectrum.00134-25 (PMC12131821; doi:10.1128/spectrum.00134-25)
Supplement: Figures S1 to S3 and Tables S1 to S3 — The results of subgroup analysis of IIM patients, disease activity of IIM patients, laboratory tests, and echocardiographic results. [file spectrum.00134-25-s0001.docx]

Supplemental Material

Supplementary Table 1. Electrocardiograph characteristics of patients with IIMs

| **Characteristic** | **No prior COVID-19 (NPC, n=72)** | **Prior COVID-19**  **(PC, n=106)** | ***p*** |
| --- | --- | --- | --- |
| Arrhythmia, n(%) | 28 (38.89) | 39 (36.79) | *0.900* |
| Malignant arrhythmia^#^, n (%) | 0 | 3 (2.83) | *-* |
| Sinus tachycardia, n (%) | 18 (25.00) | 22 (20.75) | *-* |
| Other types, n (%) | 13 (18.06) | 17 (16.04) | *-* |

# Malignant arrhythmia included ventricular fibrillation, ventricular tachycardia, ventricular arrest, third degree atrioventricular block, sick sinus syndrome, pre-excitation syndrome with atrial

**Supplementary Table 2.** **Characteristics of IIMs patients in different post-COVID periods**

| **Characteristic** | **^#^T≤3 months(n=51)** | **3＜T≤6months(n=42)** | **T＞6months(n=78)** | ***p*** |
| --- | --- | --- | --- | --- |
| MYOACT pulmonary (score) | 7.00(6.00, 8.00) | 6.00(0, 7.25) | 6.00(0,7.00) | *0.043** |
| MYOACT cardiovascular (score) | 6.00(3.00, 7.00) | 6(0.00, 6.25) | 6.00(0, 6.25) | *0.395* |
| MITAX pulmonary (score) | 9.00(3.00, 9.00) | 3.00(0, 9.00) | 3.00(0, 9.00) | *0.128* |
| MITAX cardiovascular (score) | 9.00(0, 9.00) | 9.00(0, 9.00) | 9.00(0, 9.00) | *0.708* |
| MYOACT global (score) | 0.42±0.12 | 0.35±0.11 | 0.36±0.11 | *0.004*** |
| MITAX global (score) | 0.34±0.14 | 0.29±0.12 | 0.29±0.12 | *0.045** |
| Thoracalgia, n(%) | 6(11.77) | 2(4.76) | 8(10.26) | *0.480* |
| Palpitation, n(%) | 10(19.61) | 10(23.81) | 16(20.51) | *0.874* |
| Shortness of breath/ dyspnea, n(%) | 39(76.47) | 27(64.29) | 49(62.82) | *0.243* |
| Rash, n(%) | 24(47.06) | 23(54.76) | 42(53.85) | *0.693* |
| Arthritis / arthralgia, n(%) | 13(25.49) | 12(28.57) | 21(26.92) | *0.946* |
| **Heart rate, beats per minute** | 92.02±16.76 | 91.05±14.66 | 86.83±13.94 | *0.117* |
| MYO (ng/ml) | 133.90(23.57, 759.30) | 241.50(28.58,610.50) | 115.00(30.80,596.50) | *0.872* |
| CK-MB (ng/ml) | 6.91(1.41,43.05) | 12.50(1.21,80.55) | 6.22(1.92,75.45) | *0.650* |
| cTnT (ng/L) | 44.20 (17.70,139.50) | 72.55(11.78,246.85) | 51.55(16.93,148.50) | *0.995* |
| NT-proBNP (ng/L) | 148.00(50.00,350.00) | 112.00(41.00,301.00) | 139.00(75.00,338.25) | *0.556* |
| CK (IU/L) | 212.00(44.00,1338.00) | 281.50(55.05,1525.00) | 285.50(54.75,2138.00) | *0.847* |
| LDH (IU/L) | 397.00(249.00,596.00) | 315.00(234.507.75) | 356.00(278.75,589.75) | *0.369* |
| HBDH (IU/L) | 302.00(193.00,469.00) | 245.00(176.50,402.25) | 275.00(210.50,388.25) | *0.440* |
| ALP (U/L) | 79.00(59.00,97.00) | 68.50(55.50,87.00) | 73.00(49.75,92,25) | *0.271* |
| GGT (U/L) | 61.00(19.00, 160.00) | 34.00(16.00,76.25) | 39.00(20.75,92.00) | *0.081* |
| FIB (g/L) | 2.58(2.21,3.47) | 2.87(2.43,3.42) | 2.52(2.15.3.30) | *0.254* |
| White blood cell count (×10^9^/L) | 7.52(5.06,10.15) | 7.95(5.61,10.38) | 7.36(5.86,9.44) | *0.938* |
| Neutrophils（%） | 75.60(68.88,82.00) | 70.40(59.90,80.05) | 75.60(68.00,82.00) | *0.161* |
| Neutrophils count (×10^9^/L) | 5.38(3.62,7.61) | 5.02(3.55,7.94) | 5.30(4.02,6.76) | *0.915* |
| Lymphocyte (%) | 15.90(9.40,22.20) | 18.95(12.08,25.33) | 19.00(10.83.23.50) | *0.322* |
| Lymphocyte count (×10^9^/L) | 1.14(0.77,1.64) | 1.30(0.96,2.23) | 1.31(0.80,1.89) | *0.206* |
| Platelet count (×10^9^ /L) | 193.65±59.65 | 211.64±75.86 | 206.24±79.75 | *0.443* |
| Anti-Ro52, positive, n/total (%) | 24/47(51.06) | 20/42(47.62) | 36/72(50.00) | *0.946* |
| **Clinical diagnosis myocarditis, n(%)** | 9(17.65) | 3(7.14) | 10(12.82) | *0.322* |
| LV, mm | 45.67±4.12 | 46.47±3.52 | 46.92±3.40 | *0.209* |
| LA, mm | 33.00(29.00,37.00) | 32.00(30.00,35.00) | 33.00(31.00,36.00) | *0.804* |
| RV, mm | 20.00(19.00, 22.00) | 21.00(20.00,22.00) | 21.00(19.00,23.00) | *0.126* |
| RA, mm | 32.00(30.00,34.00) | 32.00(30.00,35.25) | 33.50(31.00,37.00) | *0.052* |
| IVS, mm | 10.00(9.00, 12.00) | 9.50(8.00,11.00) | 10.00(9.00,11.00) | *0.565* |
| LVPW, mm | 9.00(8.00, 10.00) | 8.50(8.00,10.00) | 9.00(8.00,10.00) | *0.135* |
| AAO, mm | 31.50(28.25,34.00) | 32.00(29.00,35.00) | 31.00(29.00,34.00) | *0.810* |
| MPA, mm | 22.00(20.00, 23.00) | 21.50(20.00,24.00) | 22.00(20.50,24.00) | *0.846* |
| E, m/s | 0.70(0.60,0.78) | 0.70(0.60,0.90) | 0.70(0.60,0.90) | *0.077* |
| A, m/s | 0.80(0.80,1.00) | 0.80((0.70,0.90) | 0.80(0.60,1.00) | *0.992* |
| E/A≤1, n(%) | 33(64.71) | 22(52.38) | 43(55.13) | *0.425* |
| AV, m/s | 1.30(1.20,1.50) | 1.35(1.20,1.70) | 1.30(1.20,1.50) | *0.712* |
| PV, m/s | 1.00(0.80, 1.10) | 0.95(0.80,1.10) | 0.90(0.80,1.00) | *0.580* |
| e’, cm/s | 6.00(4.00,8.00) | 6.00(5.00,8.00) | 6.00(5.00,9.00) | *0.578* |
| a’, cm/s | 8.00(7.00, 9.50) | 8.50(8.00,10.00) | 8.00(7.00,10.00) | *0.470* |
| E/ e’ | 10.00(8.00, 14.00) | 12.00(9.00,12.25) | 11.00(8.00,14.00) | *0.848* |
| E/ e’＜8, n(%) | 5(9.80) | 3(7.14) | 2(2.56) | *0.212* |
| EDD, mm | 45.00(42.00, 47.75) | 47.00(43.75,48.25) | 47.00(44.75,49.00) | *0.093* |
| ESD, mm | 29.00(26.00, 30.75) | 28.00(26.00,31.00) | 29.00(26.50,32.00) | *0.605* |
| EDV, ml | 96.75±20.25 | 99.53±20.20 | 101.29±16.78 | *0.464* |
| ESV, ml | 31.00(25.00,40.50) | 29.00(25.00,39.00) | 32.00(26.00,39.00) | *0.937* |
| SV, ml | 62.07±11.15 | 67.63±11.50 | 68.05±11.77 | *0.021** |
| EF, % | 67.00(61.00,70.50) | 70.00(62.75,73.00) | 68.00(63.00,73.00) | *0.177* |
| FS, % | 37.00(33.00,42.00) | 39.00(34.00,42.00) | 38.00(34.00,42.00) | *0.339* |

*Indicates statistical difference among the 3 groups, **p*<0.05

#T means the time from improvement of COVID-19 related symptoms and negative throat swab results to admission

**Supplementary Table 3. Distribution characteristics of IIMs subtypes.**

| **Subtypes of IIMs** | **No prior COVID-19 (NPC, n=121)** | **Prior COVID-19**  **(PC, n=171)** | ***p*** |
| --- | --- | --- | --- |
| ASS | 13 (10.74) | 37(21.64) | *0.023** |
| DM (non-anti-MDA5 positive) | 35(28.93) | 32(18.71) | *0.057* |
| Anti-MDA5 positive DM | 44(36.36) | 45(26.32) | *0.088* |
| IMNM | 21 (17.36) | 32(18.71) | *0.887* |
| PM | 0 (0) | 1(0.58) | *-* |
| Unclassified IIM | 8(6.61) | 24(14.04) | *0.070* |

Data are presented as the n (%)

*Indicates statistical difference between two groups, *P<0.05.

Abbreviations: ASS, anti-synthase syndrome; DM, dermatomyositis; IMNM, immune-mediated necrotizing myopathy; PM, polymyositis; immune-mediated necrotizing myopathy;

**Supplementary Figure 1.**


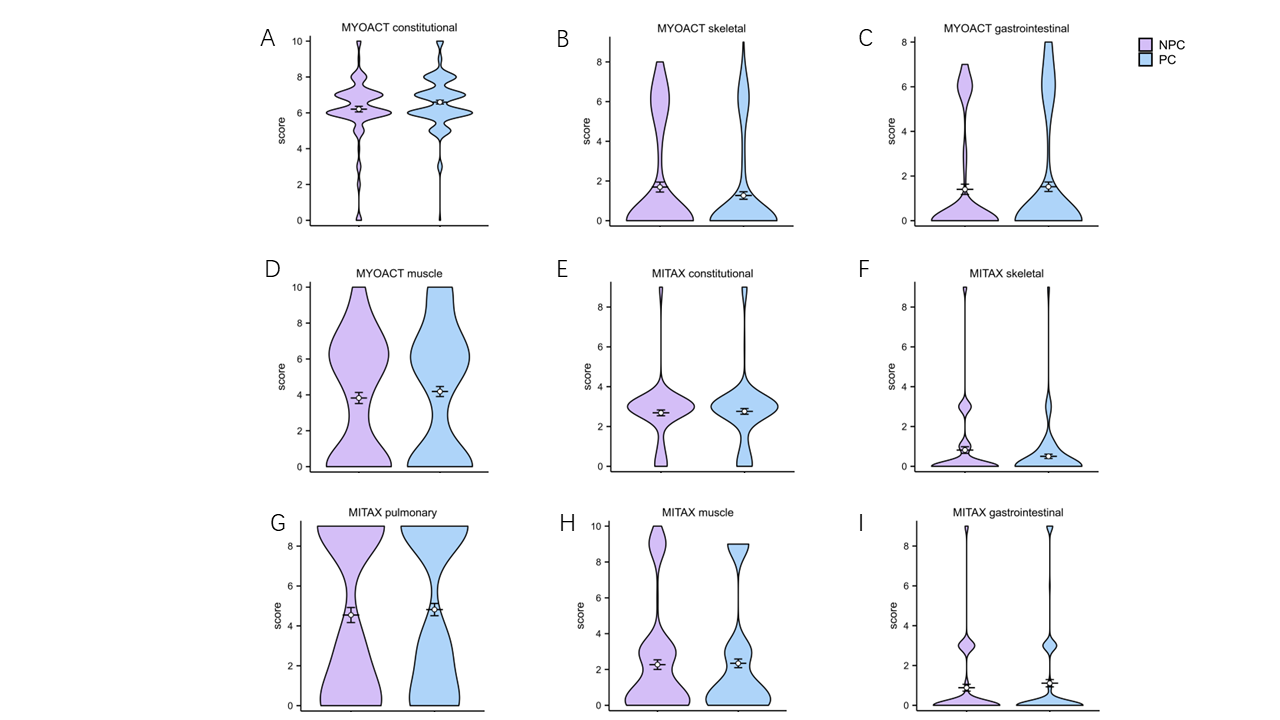


**Supplementary Figure 1. Myositis disease activity score in IIMs patients.**

MYOACT, Myositis Disease Activity Assessment Visual Analog Scales; MITAX, Myositis Intention to Treat Activity Index.

**Supplementary Figure 2.**


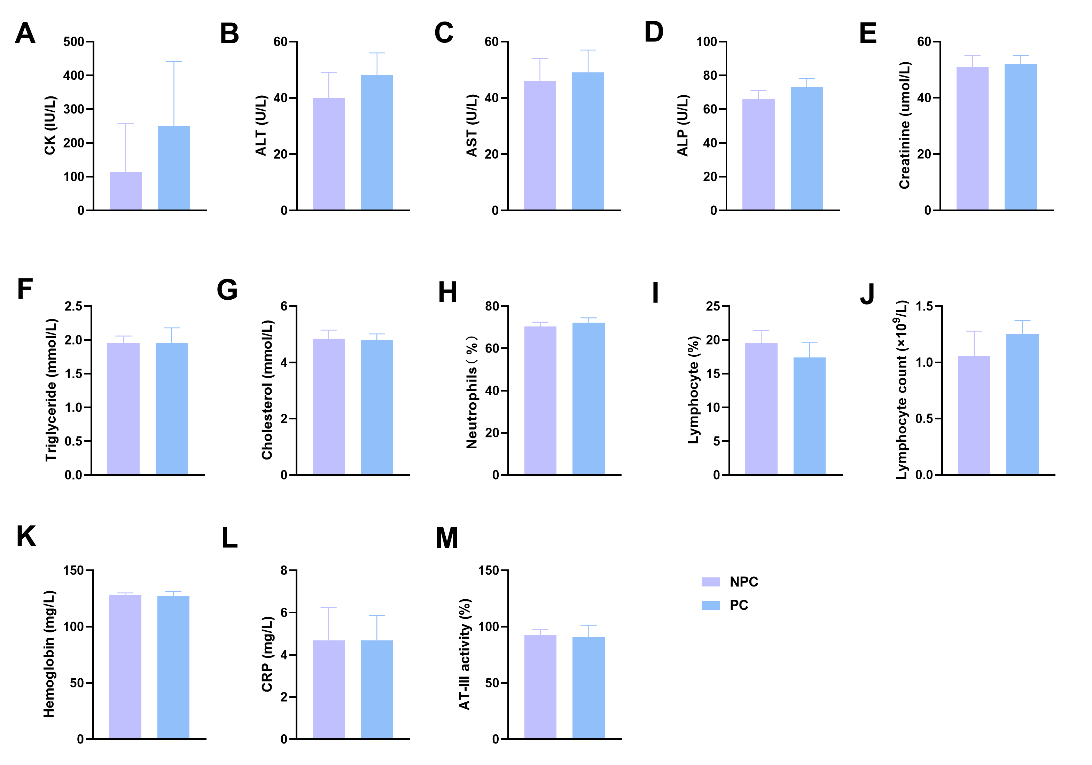


**Supplementary Figure 2. Laboratory findings of patients with IIMs.**

CK, creatine kinase; ALT, alanine transaminase; AST, aspartate aminotransferase; ALP, alkaline phosphatase; AT-III, antithrombin III; FIB, fibrinogen; CRP, C-reactive protein;

**Supplementary Figure 3**


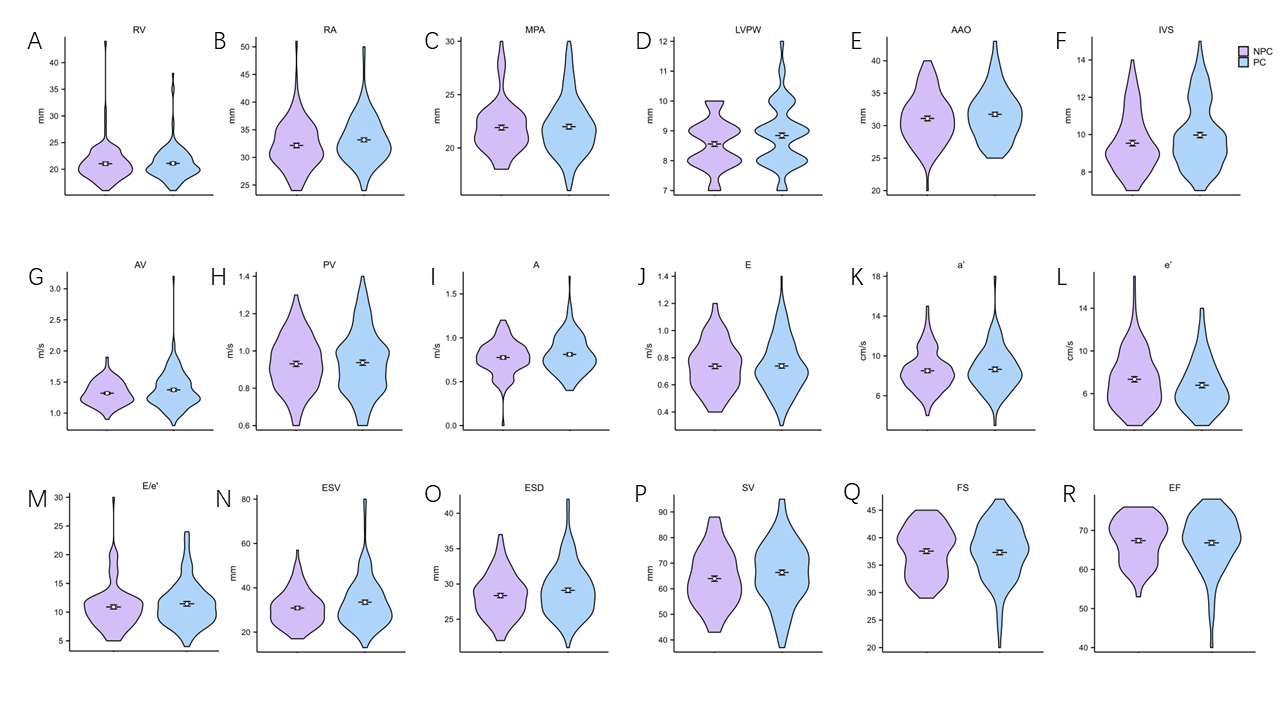


**Supplementary Figure 3. Echocardiographic characteristics of IIMs patients.**

RV, right ventricle; RA, right atrium; MPA, main pulmonary artery; LVPW, left-ventricular posterior wall; AAO, ascending aorta. IVS, interventricular sep-tum; AV, aortic valve; PV, pulmonary valve; E, peak velocity of left ventricular early-diastolic fast filling; A, peak velocity of left ventricular late-diastolic filling; e’, velocity of early diastolic myocar-dial movement at mitral ring; a’, velocity of late diastolic myocardial movement at mitral ring; ESD, end-systolic dimension; ESV, end-systolic volume; SV, stroke volume per minute; EF, ejection fraction; FS, fraction shortening;
